# Enhanced detection of prion infectivity from blood by preanalytical enrichment with peptoid-conjugated beads

**DOI:** 10.1371/journal.pone.0216013

**Published:** 2019-09-12

**Authors:** Simone Hornemann, Petra Schwarz, Elisabeth J. Rushing, Michael D. Connolly, Ronald N. Zuckermann, Alice Y. Yam, Adriano Aguzzi

**Affiliations:** 1 Institute of Neuropathology, University of Zurich, Zurich, Switzerland; 2 Novartis Vaccines and Diagnostics Inc., Emeryville, California, United States of America; 3 Biological Nanostructures Facility, The Molecular Foundry, Lawrence Berkeley National Laboratory, Berkeley, California, United States of America; University of Verona, ITALY

## Abstract

Prions cause transmissible infectious diseases in humans and animals and have been found to be transmissible by blood transfusion even in the presymptomatic stage. However, the concentration of prions in body fluids such as blood and urine is extremely low; therefore, direct diagnostic tests on such specimens often yield false-negative results. Quantitative preanalytical prion enrichment may significantly improve the sensitivity of prion assays by concentrating trace amounts of prions from large volumes of body fluids. Here, we show that beads conjugated to positively charged peptoids not only captured PrP aggregates from plasma of prion-infected hamsters, but also adsorbed prion infectivity in both the symptomatic and preclinical stages of the disease. Bead absorbed prion infectivity efficiently transmitted disease to transgenic indicator mice. We found that the readout of the peptoid-based misfolded protein assay (MPA) correlates closely with prion infectivity in vivo, thereby validating the MPA as a simple, quantitative, and sensitive surrogate indicator of the presence of prions. The reliable and sensitive detection of prions in plasma will enable a wide variety of applications in basic prion research and diagnostics.

## Introduction

Transmissible spongiform encephalopathies (TSEs) or prion diseases are caused by conformational transitioning of the physiological host-encoded prion protein, PrP^C^, into a misfolded and aggregated form, PrP^Sc^ that accumulates in the brain in the form of plaques or smaller plaque-like deposits [1, 2]. PrP^Sc^ differs from PrP^C^ in several biochemical and physicochemical properties such as its insolubility and partial resistance to proteinase K [1].

Prion diseases are prevalent in several animals and in humans. In humans, prion diseases can either appear spontaneously [3], be inherited [4] or acquired by infection [5–7] and include Creutzfeldt-Jakob disease, Gerstmann-Sträussler-Scheinker syndrome, fatal familial insomnia and Kuru. In the late 1990s a new form of CJD, designated variant CJD (vCJD) has emerged, which was traced to the transmission of prions to humans from cattle afflicted with BSE (bovine spongiform encephalopathy). The occurrence of vCJD indicated that BSE prions have overcome the species barrier between cattle and humans [8–11]. The possibility of the transmission of prions via blood was demonstrated by four confirmed fatal cases of vCJD in the UK that were acquired by blood transfusion [12, 13]. Moreover, studies performed in rodents, sheep and deer, additionally showed that prions can be efficiently transmitted via whole blood transfusion as well as buffy coats from animals in the preclinical and clinical stages of the disease [14–16].

The concentration of prions in blood, however, is low. In hamsters, where it has been studied in detail, it reaches at most 10 LD_50_ units per ml blood (LD50 represents the lethal dose where 50% of the animals inoculated with prions come down) [17, 18]. In addition, blood fractions from a single vCJD affected patient indicated an infectious titer of about 4.45 ID per mL [19]. Moreover, infectivity titers around the detection limit were found in the plasma of mice in the preclinical stage that have been infected with a mouse-adapted strain of human TSE [20].

This fact has made it difficult to utilize conventional transmission bioassays for detecting the presence of prion infectivity and has significantly hampered the validation of industrial procedures for prion removal from blood products. Assays that are based on *in vitro* amplification steps to increase the amount of detectable prions were developed [21, 22]. Efficient amplification and detection of PrP^Sc^ from the blood of hamsters infected with scrapie in the presymptomatic stage was demonstrated with the protein misfolding cyclic amplification (PMCA) assay [23]. More recently, the real-time quaking-induced conversion assay was developed, which amplifies PrP^Sc^ from tissue, cerebrospinal fluid (CSF), and other biological fluids [24, 25]. However, assays that are based on amplification steps are time-consuming and not well suited for automation and high-throughput screening applications. Also, assays that do not rely on the use of proteinase K (PK), which significantly reduces the total amount of aggregates available for detection, should allow for more sensitive detection of prions in body fluids such as blood.

An assay without additional amplification steps and PK digestion is the misfolded protein assay (MPA) [26–30]. The principle of the assay is based on a capturing step using magnetic peptoid-conjugated beads (PSR1) [26, 30, 31] that avidly and selectively bind to prion aggregates followed by quantification in an enzyme-linked immunosorbent assay (ELISA). PSR1 beads consist of magnetic beads conjugated with peptoides, which are peptidomimetics with poly-N-substituted glycines [30]. This modification makes the peptoides highly resistance to proteolytic digestion and applicable for the use in body fluidics that contain high amounts of proteases [31, 32]. The sequence of the peptoide mimics the first eight residues of the mature prion protein, KKRPKPGG. Those residues have been demonstrated to bind highly selectively to prion aggregates [26, 31]. The detection of the MPA avails on the use of the dissociation of the aggregates into monomers, thus increasing the number of available molecules and epitopes for detection in the immunoassay [30, 31]. The readouts were also found to correlate well with prion infectivity [33], suggesting that PSR1 beads physically capture prion seeds. Moreover, the MPA was able to detect PrP^Sc^, which was otherwise undetectable by proteinase K western blot analysis in the brain of a patient with familial CJD [27].

To further evaluate the reliability and sensitivity of the MPA for the detection of prion infectivity, we investigated in the present study the efficiency of PSR1 beads to capture prion infectivity from the plasma of hamsters that were in the presymptomatic and symptomatic stages of the disease. Additionally, we studied their efficiency to transmit disease to transgenic hamster indicator mice.

## Materials and methods

### Animal experimentation, animal welfare and ethics statement

All experiments with hamster were performed at Novartis Diagnostics, Emeryville, USA in accordance with their animal welfare policies. All procedures involving hamsters were approved and in compliance with the Guide for the Care and Use of Laboratory Animals by the Institutional Animal Care and Use Committee of Novartis Diagnostics, Emeryville, USA. All mice experiments were conducted in strict accordance with the Rules and Regulations for the Protection of Animal Rights (Tierschutzgesetz and Tierschutzverordnung) of the Swiss Bundesamt für Lebensmittelsicherheit und Veterinärwesen BLV. All protocols and experiments performed with mice, were specifically approved for this study by the responsible institutional animal care committee, namely the Animal Welfare Committee of the Canton of Zürich (permit number 130/2008). Mice and hamster were maintained under conventional conditions with 12h day/light cycles and all efforts were made to minimize animal discomfort and suffering. Mortality of hamster and mice that occurred outside of planned euthanasia or humane endpoints are summarized in S5 Table.

### Generation of PSR1 beads

The PSR1 beads were generated by chemical conjugation of a thiolated PSR1 peptoid derivative to magnetic beads (Dynabeads^™^ M-270 Carboxylic Acid, Invitrogen, Carlsbad CA) as described previously [30] and were provided as a 30 mg/ml suspension of beads in bead storage buffer (1xPBS with 0.1% Sodium Azide, 0.01% Triton X-100). The PSR1 peptoid loading on the beads was measured by quantitative ninhydrin assay to be 9.88 nmol/mg of beads.

### Injection of PSR1 beads into *tg*a20 mice

12 μl or 120 μl of PSR1 beads (3 μl or 30 μl beads/mouse for groups of 4 mice) were incubated overnight with brain homogenate from healthy CD1 (wt) mice (Harlan Laboratories, Netherlands). Unbound material was removed by washing five times with 1 ml PBS. Beads were resuspended in 120 μl PBS and inoculated intracerebrally (i.c.) into *tg*a*20* mice under methoxyflurane anesthesia. Tg*a*20 mice (6–8 weeks old), overexpressing the mouse prion protein [34], were randomly assigned into 2 groups: 3 mice were inoculated i.c. with 3 μl beads in a total volume of 30 μll/mouse and 4 mice with 30 μl beads/mouse. After 31 days post inoculation (dpi), mice were sacrificed under methoxyflurane anesthesia and histopathologically examined for bead accumulation and toxicity. Mice were monitored 3 times per week in accordance to the guidelines of the Animal Welfare Committee of the Canton of Zürich.

### Preparation of hamster plasma

Golden Syrian hamsters (n = 40) at one-month weanling stage were anesthetized with 3% isofluorane/L O2 and inoculated intraperitoneally (i.p.) with 100 μl of 1% (w/v) 263K hamster prion strain infected hamster brain homogenate (estimated at 10^7^ LD_50_ infectious units) using the technique outlined by Laursen and Belknap [35, 36]. Control hamsters (n = 5) were inoculated with 100 μl of a 1% non-infectious hamster brain homogenate. Blood was harvested from the hamsters at the presymptomatic stage (30, 50 and 80 dpi) and at the symptomatic stage (104–106, 117–118, 143, 154 dpi). Plasma was obtained as described previously [37]. Briefly, hamsters were anesthetized with 3% isofluorane/L O2 and blood was taken in the presence of EDTA-anticoagulant by cardiac puncture at various time points. Individual blood samples were centrifuged at 950xg for 10 minutes and the plasma in the supernatant fraction was transferred to another tube and frozen at -80°C. The clinical assessment and actions to minimize animal suffering and distress were performed as summarized in S4 Table.

### Bead-based capture of PrP^Sc^

For the sensitivity assay, groups of *Tg*(SHaPrP) mice (6–8 weeks old; n = 4–8; randomly assigned) were inoculated i.c. with 30 μl of serial ten-fold dilutions of a 263K-infected 10% brain homogenate in PBS under methoxyflurane anesthesia [38]. For the PSR1 capture assay, the plasma from 20 pre-symptomatic hamsters at 30 dpi, from 20 pre-symptomatic hamsters at 50 dpi, from 14 symptomatic hamsters at 104–106 dpi, from 15 symptomatic hamsters at 117–118 dpi, from 11 symptomatic hamsters at 143 and 154 dpi, and from 2 non-infected hamsters at 80 days inoculated with PBS were combined to individual pools. 21 μl of PSR1 beads were washed five times in 1 ml PBS (8 mM Na_2_HPO_4_, 1.5 mM KH_2_PO_4_, 137 mM NaCl, 2.7 mM KCl, pH7.4) before incubation with 500 μl of pooled hamster plasma overnight at 4°C upon shaking. Beads were again washed five times with 1 ml of PBS or TBSTT to remove unbound material and resuspended in 60 μl or 120 μl PBS, respectively. Groups of *Tg*(SHaPrP) mice (6–8 weeks old; n = 4–8, randomly assigned) were inoculated i.c. with 30 μl resuspended beads under methoxyflurane anesthesia.

For the analysis of the bead location in a *tg*a*20* mouse inoculated with RML6-coated PSR1 beads, groups of *tga*20 mice (6–8 weeks old; n = 4–8) were inoculated i.c. under methoxyflurane anesthesia with PSR1 beads treated with 10% RML6 brain homogenate ranging from 10^−2^ to 10^−10^ overnight as previously described [33]. The clinical assessment and actions to minimize animal suffering and distress were performed as laid out in S4 Table and as described previously [39].

### Histopathology and immunohistochemical stains

Two-μm thick sections were cut onto positively charged silanized glass slides and stained with hematoxylin and eosin (HE) or immunostained using antibodies for PrP (SAF84), astrocytes (glial fibrillary acidic protein, GFAP) and microglia (IBA-1). For PrP staining, sections were deparaffinized and incubated for 6 min in 98% formic acid, then washed in distilled water for 5 min.

Sections were heat-treated and immunohistochemically stained on an automated NEXES immunohistochemistry staining apparatus (Ventana Medical Systems, Switzerland) using an IVIEW DAB Detection Kit (Ventana). After incubation with protease 2 (Ventana) for 16 min, sections were incubated with anti-PrP SAF-84 (SPI bio; 1:200) for 32 min. Sections were counterstained with hematoxylin. GFAP immunohistochemistry for astrocytes (rabbit anti–mouse GFAP polyclonal antibody 1:13000 for 24 min; DAKO) and IBA-1 for microglia (rabbit polyclonal antibody 1:1000 for 24 min, WAKO) was similarly performed.

Histoblot analysis was performed using a modified standard protocol according to [40]. Briefly, 10 μm thick cryosections were mounted on glass slides and immediately pressed to a Nitrocellulose membrane (Protran, Schleicher & Schuell), soaked with lysis buffer (10 mM Tris-HCl, 100 mM NaCl, 0.05% Tween 20, pH 7.8) and air dried. After protein transfer, sections were rehydrated in TBST for 1 hour previous to Proteinase K digestion with 20, 50 and 100 μg/ml in 10 mM Tris-HCl pH 7.8 containing 100 mM NaCl and 0.1% Brij35), for 4 hours at 37 °C. After washing the membrane 3 times in TBST, a denaturation step with 3 M Guanidinium thiocyanate in 10 mM Tris-HCl, pH 7.8 was performed for 10 min at room temperature. The membrane was washed and blocked with 5% non-fat milk (in TBST) and incubated with anti-PrP antibody POM-1 (epitope in the globular domain, aa 121–231), 1:10000, overnight at 4 °C [41]. The blots were washed again and an alkaline-phosphate-conjugated goat anti mouse antibody was added (DAKO, 1:2000). Another washing step with TBST and B3 buffer (100 mM Tris, 100 mM NaCl, 100 mM MgCl_2_, pH 9.5) was followed by the visualisation step with BCIP/NBT (Roche) for 45 minutes. The colour development step was stopped with distilled water. Blots were air-dried and pictures were taken with an Olympus SZX12 Binocular and Olympus Camera.

### Immunoblotting

10% brain homogenates were prepared in 0.32 M sucrose by using a Precellys24 (Bertin). Extracts of 50–90 μg protein were digested with 50 μg/ml proteinase-K in DOC/NP-40 0.5% for 45 minutes at 37°C. The reaction was stopped by adding 3 μl complete protease inhibitor cocktail (7x concentrated) and 8 μl of a lauryl dodecyl sulfate (LDS)-based sample buffer. The samples were heated to 95°C for 5 minutes prior to electrophoresis through a 12% Bis-Tris precast gel (Invitrogen), followed by transfer to a nitrocellulose membrane by wet blotting. Proteins were detected with anti-PrP POM-1 antibody (1:10000). For secondary detection, an HRP-conjugated anti-mouse IgG antibody (Zymed, Invitrogen) was used and signals were visualized with an ECL detection kit (Pierce).

### Statistical evaluation

Results are expressed as the mean ± standard deviation (SD) as indicated.

## Results

### Assessment of bead toxicity

**Fig 1:**
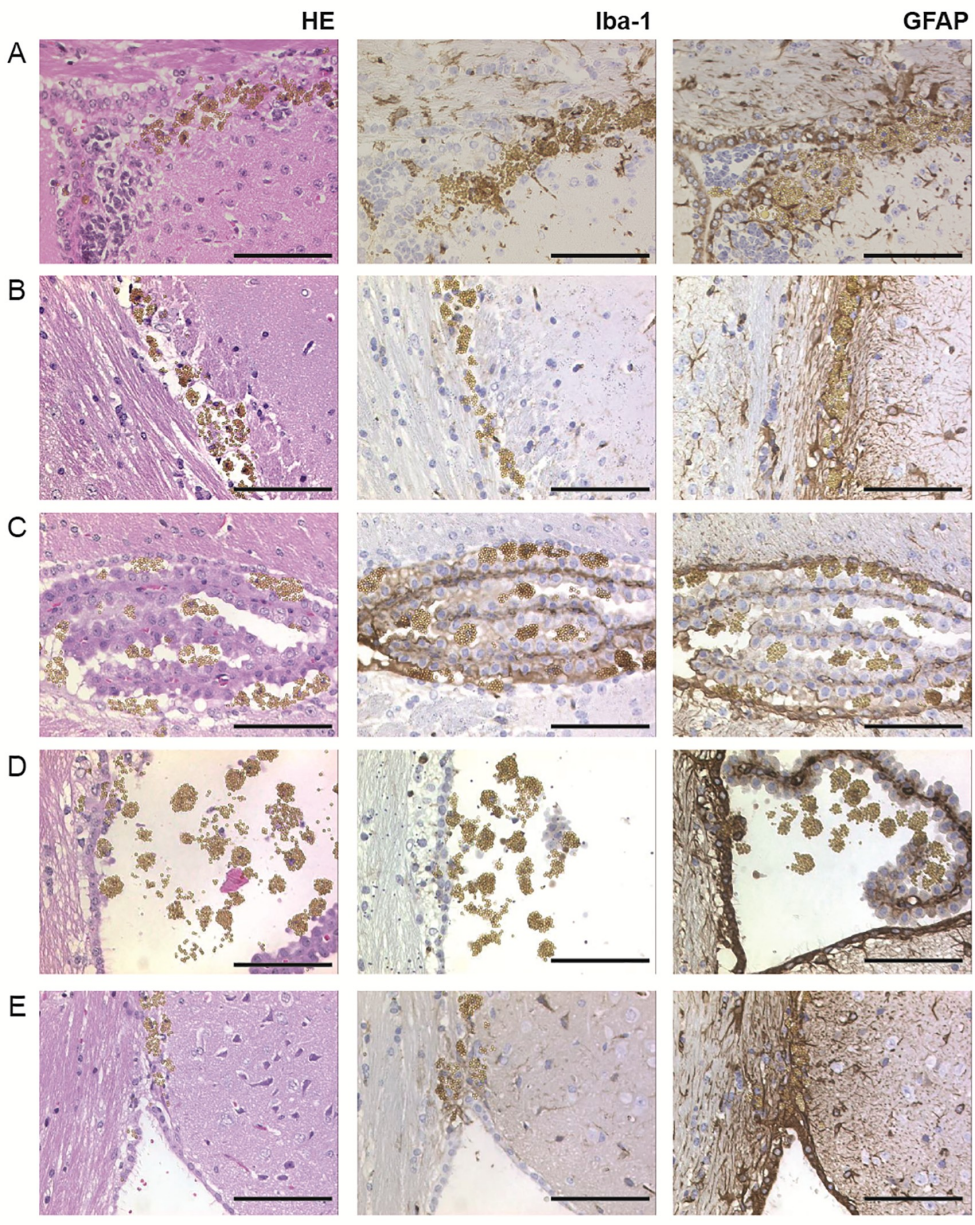
Histopathological analysis of tissue reactions to intracerebral bead inoculation. *Tg*a*20* mice were inoculated with PSR1 beads that had been exposed to wt CD1 brain homogenates. Vacuolation by hematoxylin-eosin staining (HE), astrocytic gliosis was documented by an antibody directed against the glial fibrillary acidic protein (GFAP), and microgliosis was assessed with the activated microglial marker (Iba-1). (A) Brain sections of a *tg*a*20* mice analysed at 8 dpi showed bead accumulation near the injection site. (B) Brain sections analyzed at 31 dpi showed accumulation of the beads adjacent to the corpus callosum, (C) choroid plexus and (D) lateral ventricle, and in the area between corpus callosum and the septofimbrial nucleus (E). (Scale bars: 100 μm).

We first evaluated in a pilot experiment, whether peptoid-conjugated beads exposed to brain homogenates from healthy wild-type CD1 mice would induce any acute toxic response in recipient mice (Table 1). 3 μl or 30 μl beads, respectively, from a 30 mg/ml suspension of beads were incubated overnight with the brain homogenate and inoculated i.c. into groups of 3 and 4 *tg*a*20* mice, respectively, overexpressing the mouse prion protein [42]. All mice were sacrificed at 31 days, except for one mouse that had already died at 8 dpi, and subsequently analysed for acute toxicity and bead accumulation. Beads were detectable only at the injection site in the brain of the mouse, that had died at 8 dpi, (Fig 1A, S1 Fig), whereas in the remaining mice they were found to be broadly distributed in both hemispheres. Beads were often observed to be grouped in small clusters and were more abundant in intra- and periventricular regions, including the corpus callosum, ependymal lining, choroid plexus as well as the leptomeninges (Fig 1B–1E, S2 and S3 Figs). Mild gliosis and microglial activation were observed around the injection site at 8 dpi (Fig 1A), while a more diffuse astrogliosis was observed at 31 dpi (Fig 1B–1E, S2 and S3 Figs). Gliosis, which is a nonspecific reaction to injury in the central nervous system [43], was more pronounced in the hippocampus, deep gray nuclei, medial habenular nucleus, brainstem and deep cerebellar white matter. As the lateral ventricles did not show evidence of hydrocephalus, we conclude that the beads did not significantly disturb the outflow of the cerebrospinal fluid (S1 Fig).

**Table 1:** Pilot experiment: Assessment of bead toxicity in *tga20*.

PSR1 beads, coated with healthy brain homogenate	Total inoculated	Sacrificed at
3 μl beads in 30 μl PBS/mouse	3	31, 31, 31 dpi
30 μl in 30 μl PBS beads/mouse	4	8*, 31, 31, 31 dpi

*Mouse died of an intercurrent death.

### End-point titration of the 263K hamster strain in *Tg*(SHaPrP)

**Fig 2:**
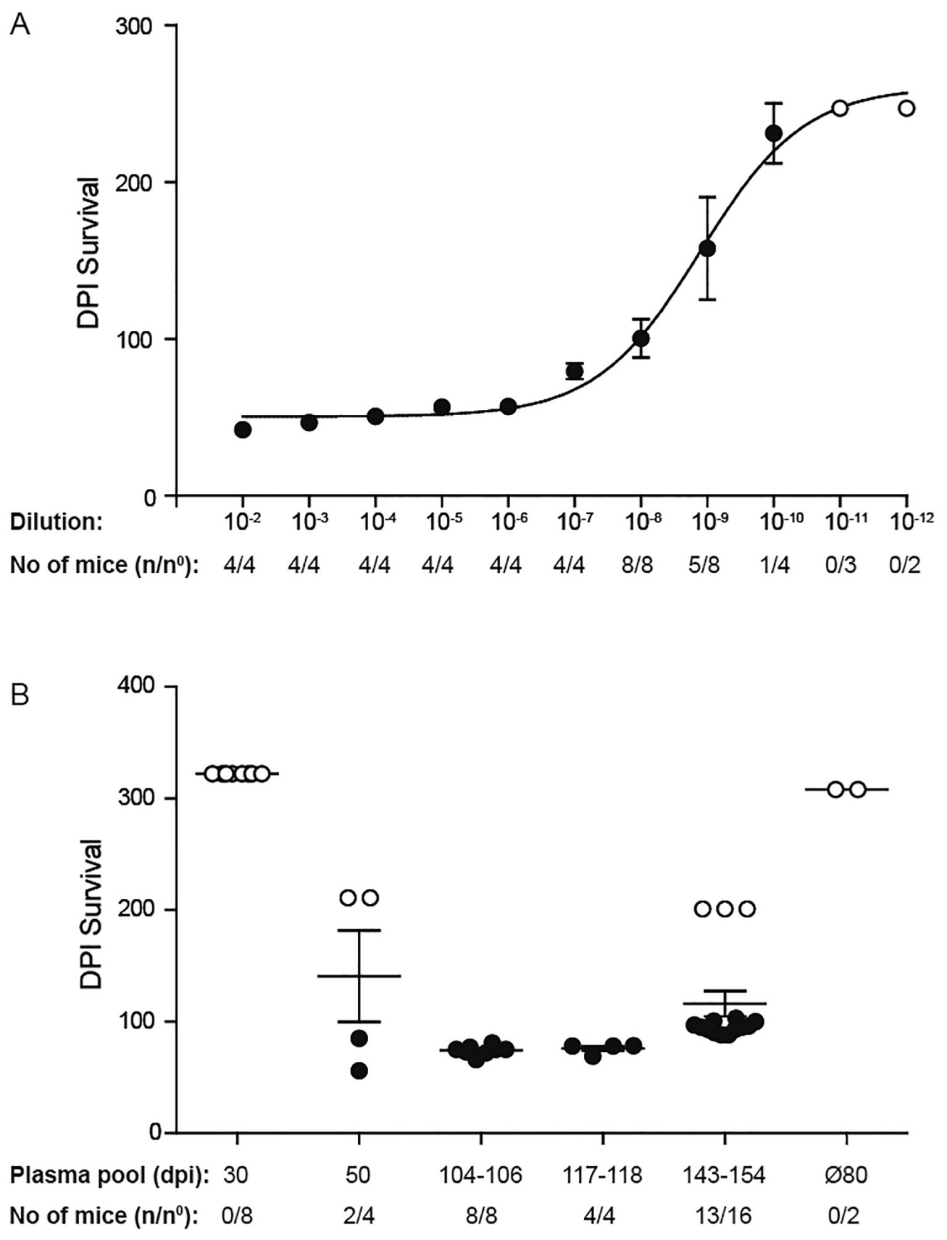
Survival plots of *tg*(SHaPrP) mice. **(A)** For titer determination, *tg*(SHaPrP) mice were inoculated with serial 10-fold dilutions of a 10% (wt/vol) 263K hamster brain homogenate ranging from 10^−2^ to 10^−12^. **(B)** Bioassay of *Tg*(SHaPrP) mice that were inoculated i.c. with PSR1 beads incubated with pooled plasma samples from hamsters infected with 263K prions. Individual pools of plasma samples from pre-symptomatic hamsters harvested at 30 (n = 20) and 50 dpi (n = 20) and from symptomatic hamsters harvested at 104–106 dpi (n = 14), 117–118 dpi (n = 15), and 143 and 154 dpi (n = 11) were used. Data points: mean incubation times ± standard error of the mean. Mice that did not develop a prion disease until the end of the experiment are indicated by open circles. n/n°: indicates the attack rate (number of mice developing a prion disease divided by the total number of inoculated mice). Ø80: indicates the control group of mice inoculated with PSR1 beads after incubation with plasma from healthy 80 days old hamsters (n = 2).

**Fig 3:**
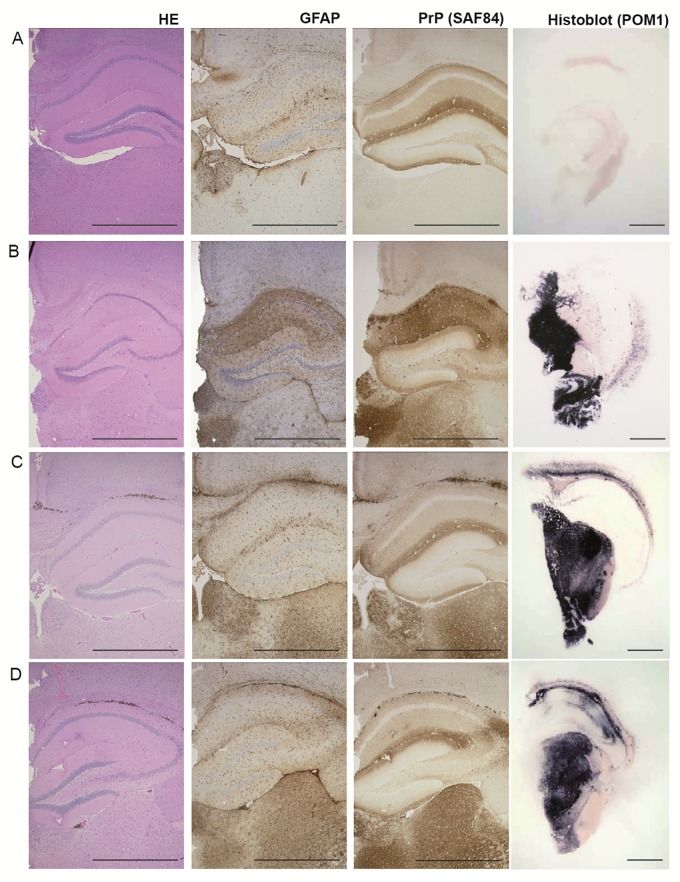
Pathology of hippocampus, medial habenular and thalamic nuclei from *Tg*(SHaPrP) mice. Non-inoculated mice **(A)** show no evidence of vacuolation, PrP^Sc^ deposition or gliosis. Mice inoculated with 263K prion-infected hamster brain homogenate **(B)**, inoculated with PSR1 beads incubated with plasma pools from symptomatic hamster at 117–118 dpi **(C)** and at 143 and 154 dpi **(D)** show vacuoles in the HE stained section (see also S2 and S3 Figs), PrP^c^ and PrP^Sc^ deposition as visualized by the PrP antibody SAF84 and astrocytic gliosis as evidenced by an antibody directed against GFAP. Histoblot analysis was used to show PrP^Sc^ deposition after proteinase K digestion and staining with POM1. (Scale bars: 1 mm).

10-fold serial dilutions (10^−2^ to 10^−12^) of a 10% (w/v) 263K hamster brain homogenate were i.c. inoculated into groups of 4 to 8 *Tg*(SHaPrP) mice, overexpressing the wild-type hamster prion protein [38] (Fig 2A, Table 2). Clinical signs were observed in mice inoculated with dilutions spanning 10^−2^ to 10^−9^ after mean incubation periods between 40 and 107 days (Fig 3, Table 2). A standard calibration curve for the infectivity of the 263K hamster strain in *Tg*(SHaPrP) was generated and a median 50% lethal dose [LD_50_] of 10^9.23^ units ml^-1^ was calculated using the Reed-Muench formula [44–47].

**Table 2:** Summary of end-point titrations of the 263K inoculum in *Tg*(SHaPrP).

Dilution of brain homogenate^a^	(Clinical TSE/total inoculated)	Mean incubation period (days ± SD) [42, 47]
10^−2^	4/4	42 ± 1
10^−3^	4/4	47 ± 1.4
10^−4^	4/4	51 ± 0.5
10^−5^	4/4	56 ± 1.2
10^−6^	4/4	57 ± 3.3
10^−7^	4/4	79 ± 7.4
10^−8^	8/8	100 ± 34
10^−9^	5/8	83, 90, 90, 90, 107, >250, >250, >250
10^−10^	1/4	174^b^, >250, >250, >250
10^−11^	0/3	>247
10^−12^	0/2	>247

^a^ Dilutions were started from a 10% brain homogenate.

^b^ The incubation period to terminal disease was about 174 days at the end-point. An approximate linear regression curve (y = 10.6–0.105x; r = 0.87) was calculated from the mean incubation periods of dilutions 10^−2^ to 10^−9^.

### Bioassay in *Tg*(SHaPrP) mice of PSR1 beads treated with plasma from prion infected hamster

Next, we determined in a bioassay in Tg(SHaPrP) transgenic mice, whether the PSR1 beads were able to capture quantitatively prion infectivity from plasma samples. To that purpose, we used pools of plasma from 263K prion-infected hamster at presymptomatic or symptomatic stages. Plasma samples from symptomatic hamsters were collected at the stage of onset of clinical signs showing the typical disease characteristics of ataxia, loss of appetite and poor grooming. The individual pools (Table 3) were incubated overnight with the PSR1 beads. After an intense washing step, the beads were i.c. inoculated into Tg(SHaPrP) mice [38]. None of the mice inoculated with PSR1 beads coated with plasma from presymptomatic hamsters (30 dpi) nor any of the negative control mice showed any clinical signs of a prion disease. However, two mice (n = 4) inoculated with beads incubated with plasma from a presymptomatic hamster (50 dpi) developed disease with highly variable incubation periods after 56 and 85 dpi (equivalent to an apparent ID_50_ of 3.2 ± 2.2 units ml^-1^ of 30 μl 10% 263K hamster brain homogenate, Fig 2B, Table 3) and incomplete attack rate.

**Table 3:** Summary of bioassays with *Tg*(SHaPrP) mice that were inoculated with plasma coated PSR1 beads.

Plasma Pools	Attack rate (Clinical TSE/total inoculated)	Mean incubation period (days)	Estimated titers* Log LD_50_ units ml^-1^ [42, 47]
pre-symptomatic hamster, 20 animals, 30 dpi	0% (0/8)	8 survivor terminated at 322 dpi	
pre-symptomatic hamster, 20 animals, 50 dpi	50% (2/4)	56 and 85 dpi (71 ± 21 dpi) (plus 2 survivor terminated at 211 dpi)	3.2 ± 2.2
symptomatic hamster, 14 animals, 104–106 dpi	100% (8/8)	74 ± 3 dpi	2.8 ± 0.3
symptomatic hamster, 15 animals, 117–118 dpi	100% (4/4)	76 ± 5 dpi	2.4 ± 0.5
symptomatic hamster, 11 animals, 143 dpi and 154 dpi	81% (13/16)	94 ± 5 dpi (plus 3 survivor terminated at 211 dpi)	1.9 ± 0.5
non-infected hamster, 2 animals, 80 days after PBS inoculation	0% (0/2)	8 survivor terminated at 308 dpi	

*Titers were estimated from the calibration curve of the end-point titrations of the 263K inoculum in *Tg*(SHaPrP) (Table 2; Fig 2) using the incubation time method.

Most other mice inoculated with beads treated with the different plasma pools from symptomatic hamster showed mean incubation periods between 74 ± 3 and 94 ± 5 dpi (equivalent to apparent ID_50_’s of 2.8 ± 0.3 to 1.9 ± 0.5 units ml^-1^ Fig 2B, Table 3). The incubation times within the transgenic mouse bioassay increased with the use of inoculums obtained from symptomatic hamster with longer incubation times. The longest incubation time in the indicator mice was observed for the pooled inoculum derived from symptomatic hamster sacrificed at days 143 dpi and 154 dpi. This group of mice also exhibited a lower attack rate of 81%.

**Fig 4:**
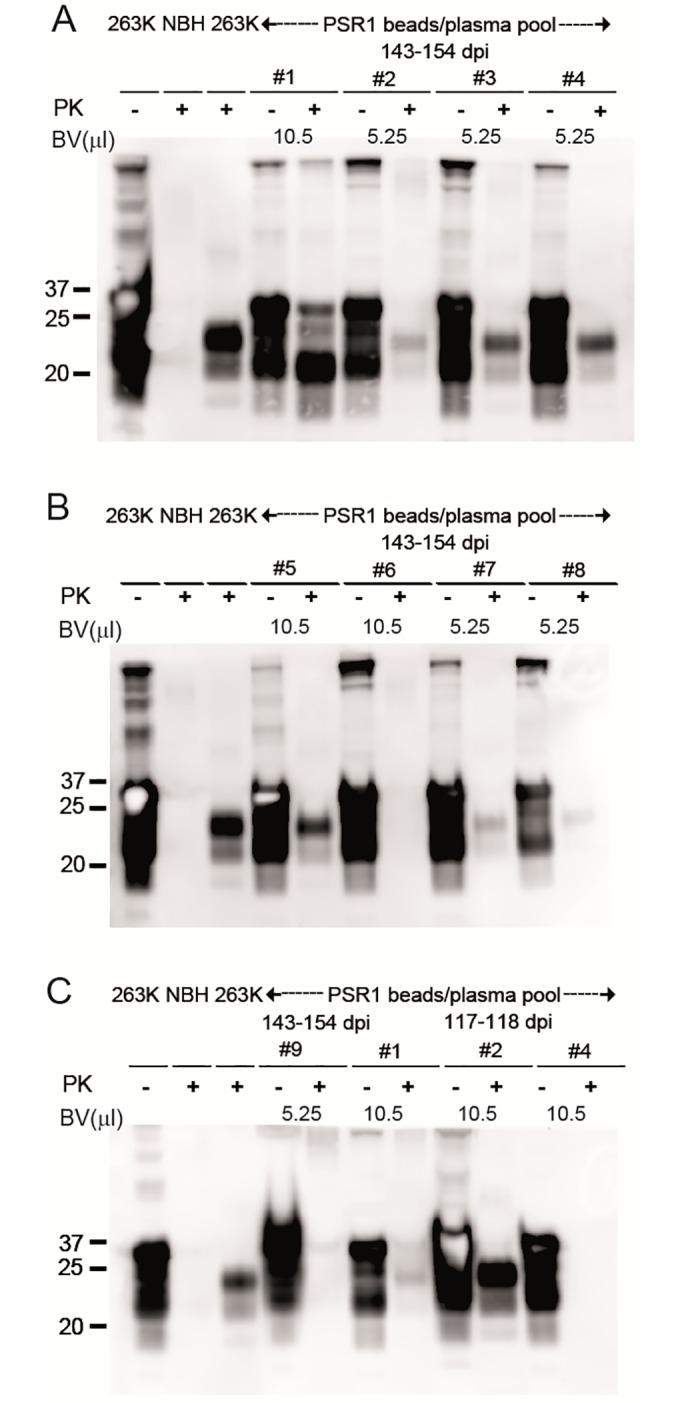
PK-Western blot analysis of brain homogenates from *Tg*(SHaPrP) mice inoculated with PSR1 beads coated with plasma from symptomatic hamster. (A-C) PrP^Sc^ is present in *Tg*(SHaPrP) inoculated i.c. with PSR1 beads incubated with plasma pools from symptomatic hamsters. Mice were incubated either with 5.25 or 10.5 μl of PSR1 beads in PBS (A) or in TBSTT (B-C). Because of the high expression level of PrP^C^ in *Tg*(SHaPrP), PrP^C^ is still detectable on Western blots. Control samples are labeled with NBH: non-infected brain homogenate from healthy mice, and 263K: brain homogenate from mice inoculated with 263K prions. BV: bead volume. The anti-PrP antibody POM-1 was used for detection. The molecular weight standard is shown in kilodaltons.

The calculated titers are slightly higher than the apparent titer of 1.25 LD_50_ ml^-1^, estimated from the quantity of 10 LD_50_ ml^-1^ infectivity found in the blood of hamsters during the symptomatic stage of the disease [17, 48], for 30 μl resuspended PSR1 beads coated with plasma from prion-infected hamster. Histopathological and immunohistochemical analysis of the brains of the mice was used to diagnostically confirm the presence for a prion disease (Fig 3). Additionally, PK resistant PrP, a surrogate marker of prion disease, could be detected by histoblot and Western blot analyses (Figs 3 and 4). We therefore conclude that PSR1 beads highly efficiently capture prion infectivity from plasma from presymptomatic and symptomatic cases and are able to transmit infectivity to *Tg*(SHaPrP) mice.

Mice were also analyzed after termination for the location of the beads. After 90 dpi, beads were mostly found in the brain, spinal cord and CSF, but also in most other organs (if only in small numbers) as well as in urine and feces (see S1–S3 Tables). After 250 dpi, only a few beads were observed in the brain with occasional beads around the spinal cord, in the spleen and in selected other organs (see S1–S3 Tables). A similar distribution of the beads was also observed in *tga*20 mice i.c. inoculated with PSR1 beads treated with a 10^−9^ dilution of a 10% (w/v) RML6 brain homogenate and sacrificed at 253 dpi (S4 Fig, [33]). These data indicate that beads might have been transported along the axonal route [49] or lymphatics [50, 51].

## Discussion and conclusion

Previously, we have reported that the MPA could detect prion aggregates with high sensitivity in the brain of a patient with a familial prion disease [27]. Crucially, the MPA detected PK sensitive conformers of PrP^Sc^ that were only barely detectable by standard methods [27]. The analytical sensitivity of the MPA has recently been determined in a spiking experiment of a BH from a vCJD patient into normal human plasma. PrP aggregates were still detectable at a dilution below 10 nl/ml (10^−6^ dilution, LOD estimated to be < 40 pg/ml of aggregated PrP) [31]. These findings prompted us to investigate further the sensitivity of the peptoid-conjugated beads to capture prion infectivity from the plasma of prion-infected hamsters. This is biologically and medically relevant because (1) the conformation of infectious PrP in blood is unknown and (2) the detection of prions in blood and blood products continues to represent a major analytical challenge.

Our data showed that the PSR1 beads were able to enrich low concentrations of infectivity from the plasma of hamsters in symptomatic and even presymptomatic stages of the disease. Although we have not performed a direct injection of plasma pools from 263K prion-infected hamster at presymptomatic or symptomatic stage to estimate the infectivity titers, our findings suggest that the beads are capable of capturing prion infectivity from plasma at concentrations around the threshold of detection identified earlier in rodent prion infectivity experiments for presymptomatic cases [17, 18]. Alternatively, the high capture efficiency of PSR1 beads could stem from an ability to detect more PK sensitive conformations of PrP^Sc^, as prions in plasma were proposed to consist of more PK sensitive conformations of PrP^Sc^ [52]. The apparent higher titers of PrP^Sc^ on the bead surfaces might also be caused from PrP^Sc^ being protected against degradation on the bead, e.g. by limiting exposure to proteolysis.

Transmission of infectivity from solid-state surfaces might occur either by dissociation of infectivity or by the transport of these surfaces to different brain areas where they initiate infection [53, 54]. We observed that the beads stayed at the injection site within the first days after injection. After 31 days, the beads were found to be more diffusely distributed within the brain, with a greater density in periventricular regions and within the leptomeninges. After 90 days, only a few beads could be detected, which were subsequently found in most organs and body fluids. Possible routes of clearance include the internal and external veins that enter the dural sinuses, and then eventually drain into the internal jugular veins. Other potential conduits for clearance are along axons [49] or recently described lymphatic vasculature that lines dural sinuses in the meninges and drains into cervical lymph nodes [50, 51, 55].

Prions have been shown to form structurally distinct PrP^Sc^ conformers, known as prion strains [56]. Those strains consist of different particle size distributions of prion aggregates responsible for different biophysical properties that determine the course of the disease [57]. The size of the PrP aggregate might influence PSR1 avidity and impair the sensitivity of the assay for strains composed of smaller prion aggregates. However, PSR1 beads were shown to bind to a broad range of Abeta amyloidogenic aggregates, the key species in the pathology of Alzheimer’s disease (AD) [58], with different conformations and of different sizes ranging from at least <50 to >400 amino acids [30]. This gives evidence that PSR1 might bind sensitively to a broad range of strains that are composed of aggregates of different size.

The ability of PSR1-coated beads to efficiently capture and enrich prions from plasma directly suggests the feasibility of solid-state prion-capture matrices for both clinical and research uses. For example, the beads might be applied for depletion, removal and clearance of prions from biological fluids and biopharmaceutical products. The number of beads can easily be increased and their magnetic properties should allow the capture of prions even in high volumes. In a further embodiment, the beads might also be applied in concert with the scrapie cell endpoint assay (SCEPA) [59] and/or with prion amplification assays, such as the PMCA and RT-QuIC [60, 61]. Pre-analytical capture has recently been shown to improve the sensitivity of these assays by several orders of magnitude, enabling the sensitive and selective detection of prions in blood and body fluidics from various species [59–61], including those from vCJD patients [62, 63]. Different methods have been applied for pre-analytical capture, including the capture with magnetic beads containing a Fe_3_O_4_ metal surface [60, 64], solid-state capture matrices consisting of stainless steel [63] or magnetic plasminogen coated-beads [62, 65]. Magnetic Fe_3_O_4_ metal surface beads [60, 64] have been described to avidly bind to the metal-binding domains of aggregated PrP [66, 67]. Similarly, the avidly binding of stainless steel surfaces to PrP aggregates is mediated by an interaction to nickel and molybdenum [68]; most properly also induced by the metal-binding domains of PrP. Finally, the binding of plasminogen coated magnetic nanobeads to PrP aggregates has been reported to be based on specific interactions of the lysine binding site 1 of plasminogen [69]. The binding mode of these capture reagents have in common with the one of the PSR1 beads that they are based on the property of PrP aggregates to avidly bind to various surfaces using non-covalent binding interactions [53, 54]. The interaction of PSR1 beads to PrP aggregates relies on the cooperative binding of cationic PSR1 residues to negatively charged PrP aggregates [26, 30, 31], whereas the interaction with monomeric PrP is weak [26, 30, 31]. This allows the selective capture of PrP aggregates with an about 4000-fold higher binding specificity for aggregated PrP over monomeric PrP [26]. Multivalent non-covalent interaction-based immobilization procedures therefore seem to be highly suitable for the efficient capture and enrichment of PrP aggregates that are only present at very low quantities in blood.

Finally, the PSR1 beads might also be used together with a sensitive detection method to diagnose prions. Prion bioassays report the infectious titer and do not directly reflect the total number of PrP molecules, whereas the MPA reports the amount of PrP in aggregated conformations. Because each aggregate can be composed of a vast number of PrP molecules, the subsequent disaggregation can lead to the exposure of many epitopes for sensitive immunodetection. A sensitive prion detection assay based on the MPA technology could be easily automatable and optimized for high-throughput applications, eventually paving the way to early and sensitive blood diagnosis of prion diseases in humans and to the prevention of transmission of prions through economical and reliable screening of blood and blood products.

## Supporting information

S1 ARRIVE Guidelines checklist. (PDF)

S1 Fig Histopathological analysis of tissue reactions to intracerebral bead inoculation. Coronal sections at low magnification show that the outflow of the CSF through the lateral ventricles is not affected. **(A)** Brain sections of a *tg*a*20* mouse inoculated with PSR1-beads scarified at 8 dpi. **(B)** Brain sections of a *tg*a*20* mouse inoculated with PSR1-beads and terminated at 31 dpi. Vacuolation was again visualized by HE staining, astrocytic gliosis by staining the GFAP protein with a GFAP-specific antibody, and microgliosis by the activated microglial marker Iba-1. Activation of astrocytic gliosis and microgliosis was detected near the injection. (Scale bars: 1 mm).
(TIF)

S2 Fig Same as in Fig 3, but the pathology of sections of the hippocampus and corpus callosum are shown at higher magnification to better visualize vacuolation. Non-inoculated mice **(A)** show no evidence of vacuolation, PrP^Sc^ deposition or gliosis. Mice inoculated with 263K prion-infected hamster brain homogenate **(B)**, inoculated with PSR1 beads incubated with plasma pools from symptomatic hamster at 117–118 dpi **(C)** and at 143 and 154 dpi **(D)** show vacuoles in the HE stained section, PrP^c^ and PrP^Sc^ deposition is visualized by the PrP antibody SAF84 and astrocytic gliosis is evidenced by an antibody directed against GFAP. (Scale bars: 100 μm).
(TIF)

S3 Fig Same as in Fig 3, but the pathology of sections of the thalamus are shown at higher magnification to better visualize vacuolation. Non-inoculated mice **(A)** show no evidence of vacuolation, PrP^Sc^ deposition or gliosis. Mice inoculated with 263K prion-infected hamster brain homogenate **(B)**, inoculated with PSR1 beads incubated with plasma pools from symptomatic hamster at 117–118 dpi **(C)** and at 143 and 154 dpi **(D)** show vacuoles in the HE stained section, PrP^c^ and PrP^Sc^ deposition is visualized by the PrP antibody SAF84 and astrocytic gliosis is evidenced by an antibody directed against GFAP. (Scale bars: 100 μm).
(TIF)

S4 Fig Histopathological analysis of bead location in tissue sections from a *tg*a*20* mouse inoculated with RML6-coated PSR1 beads. The mouse shown was inoculated with RML6 brain homogenate (10^−9^ dilution) and euthanized at 253 dpi. HE stained sections of brain, spleen, spinal cord ganglion and spinal cord out nerve are shown. Beads are indicated with yellow arrows. (Scale bars: 50 μm).
(TIF)

S5 Fig Uncropped and unmodified Western blots of Fig 4. (TIF)

S1 Table Location of PSR1 beads in body fluids from mice inoculated with 3 μl beads coated with plasma from prion-infected hamster per mouse. Yes: beads were present in these body fluids. No: no beads were observed. n.a.: non-analyzed. dpi: days post infection. The analysis was only performed for the indicated mice.
(PDF)

S2 Table Presence of PSR1 beads in various tissue homogenates from mice inoculated with 3 μl beads coated with plasma from prion-infected hamster per mouse. Yes: beads were found in these homogenates. No: no beads were observed. n.a.: non-analyzed. dpi: days post infection. The analysis was only performed for the indicated mice.
(PDF)

S3 Table Presence of PSR1 beads in various organs from mice inoculated with 3 μl beads coated with plasma from prion-infected hamster per mouse. Paraffin sections of various organs were analysed for the presence of the beads. Yes: beads were found in these organs. No: no beads were observed. n.a.: non-analyzed.?: not clearly identified. BM: bone marrow. dpi: days post infection. The analysis was only performed for the indicated mice.
(PDF)

S4 Table Clinical assessment and scoring of Golden Syrian hamsters inoculated with the 263K hamster prion strain, *tg*a20 mice inoculated with RML6, and *Tg*(SHaPrP) mice either inoculated with 263K or with plasma coated PSR1 beads as described in [39]. Animals were monitored 3 times per week after RML6 or 263K inoculation for clinical signs including gait, grooming, activity, rough hair coat, limb paresis and ataxia. Once the animals showed the first sign of scrapie (grade 1), they were monitored every day and wet food was supplied in the cage. When the animals reached score grade 2 that hindered them reaching the water bottle, they were either euthanized with 3% isofluorane/L O2 (hamster) or methoxyflurane (mice), followed by decapitation.
(PDF)

S5 Table Mortality of hamster and mice that occurred outside of planned euthanasia or humane endpoints. (PDF)

## Abbreviations

ELISA: Enzyme-Linked Immunosorbent Assay
MPA: Misfolded Protein Assay
mPrP: mouse PrP
PrP: prion protein
PrP^C^: cellular isoform of PrP
PrP^Sc^: scrapie isoform of PrP^C^
PSR1: Prion Specific Reagent 1
RML6: Rocky Mountain Laboratory strain, passage 6
TSE: transmissible spongiform encephalopathy
