# Supplementary figures and images for: Enhanced detection of prion infectivity from blood by preanalytical enrichment with peptoid-conjugated beads
Source: PLoS One. 2019 Sep 12;14(9):e0216013. doi: 10.1371/journal.pone.0216013 (PMC6742390; doi:10.1371/journal.pone.0216013)

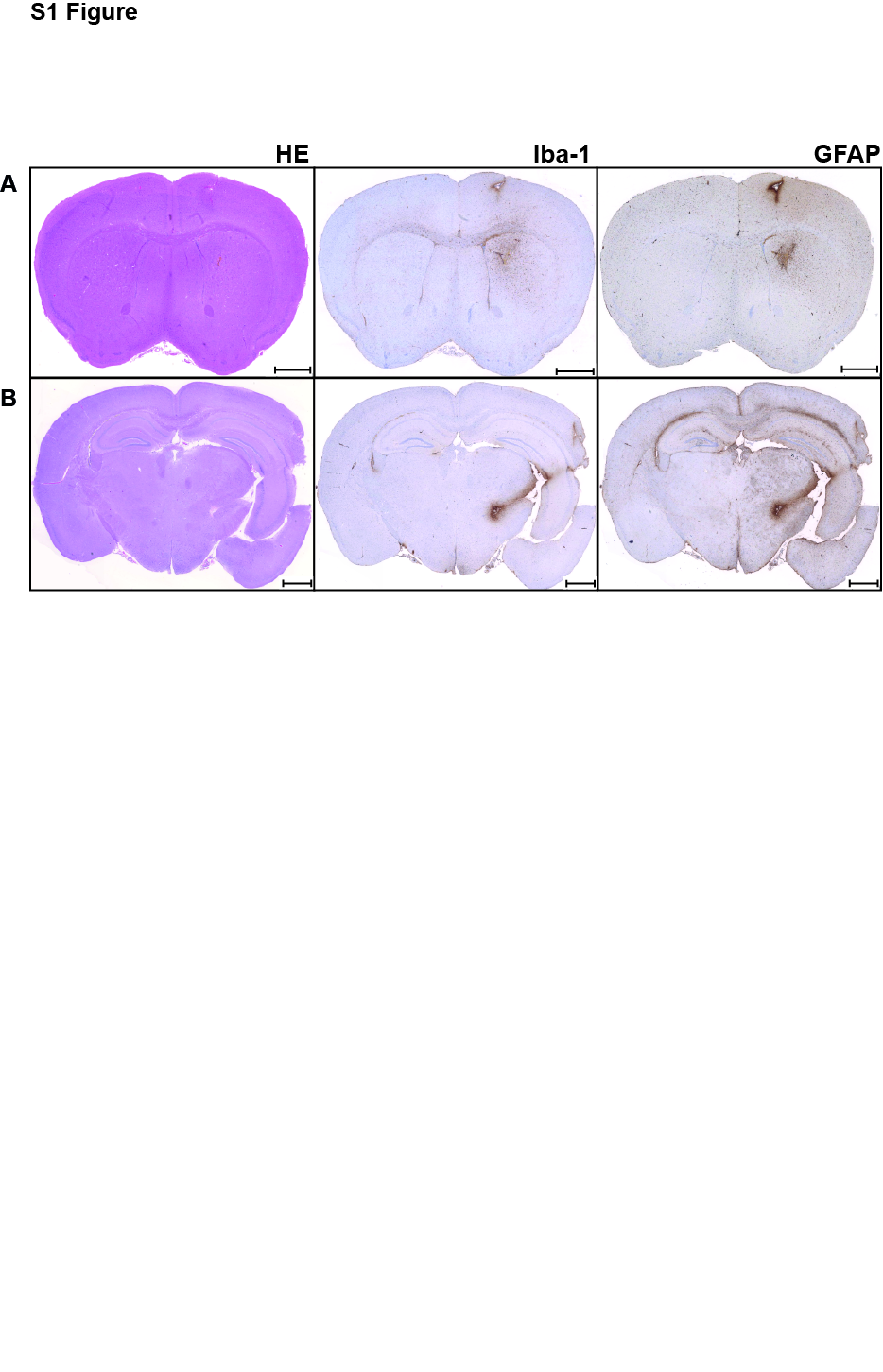

Supplement: S1 Fig — Coronal sections at low magnification show that the outflow of the CSF through the lateral ventricles is not affected. (A) Brain sections of a tga20 mouse inoculated with PSR1-beads scarified at 8 dpi. (B) Brain sections of a tga20 mouse inoculated with PSR1-beads and terminated at 31 dpi. Vacuolation was again visualized by HE staining, astrocytic gliosis by staining the GFAP protein with a GFAP-specific antibody, and microgliosis by the activated microglial marker Iba-1. Activation of astrocytic gliosis and microgliosis was detected near the injection. (Scale bars: 1 mm). (TIF) [file pone.0216013.s002.tif]

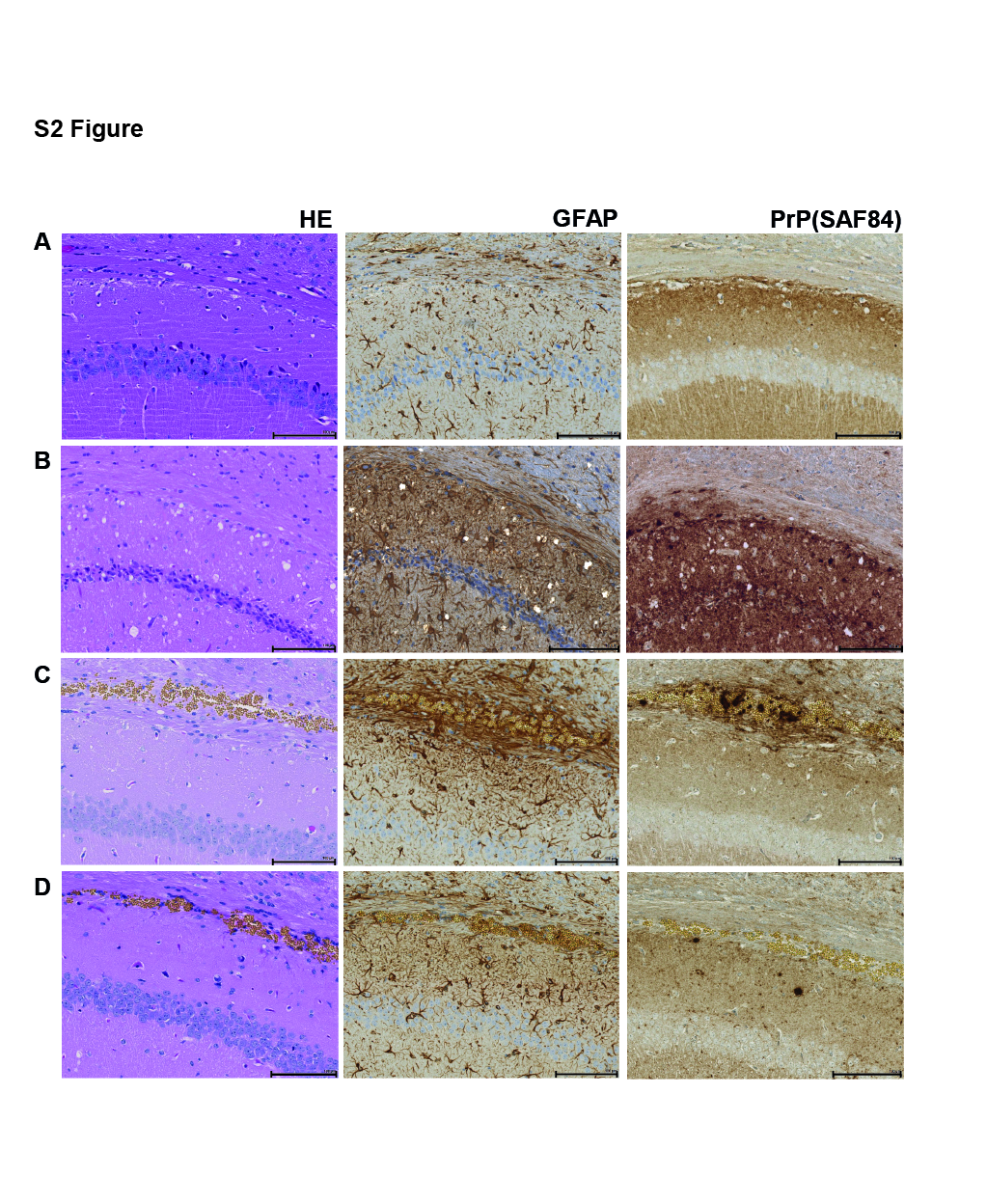

Supplement: S2 Fig — Non-inoculated mice (A) show no evidence of vacuolation, PrPSc deposition or gliosis. Mice inoculated with 263K prion-infected hamster brain homogenate (B), inoculated with PSR1 beads incubated with plasma pools from symptomatic hamster at 117–118 dpi (C) and at 143 and 154 dpi (D) show vacuoles in the HE stained section, PrPc and PrPSc deposition is visualized by the PrP antibody SAF84 and astrocytic gliosis is evidenced by an antibody directed against GFAP. (Scale bars: 100 μm). (TIF) [file pone.0216013.s003.tif]

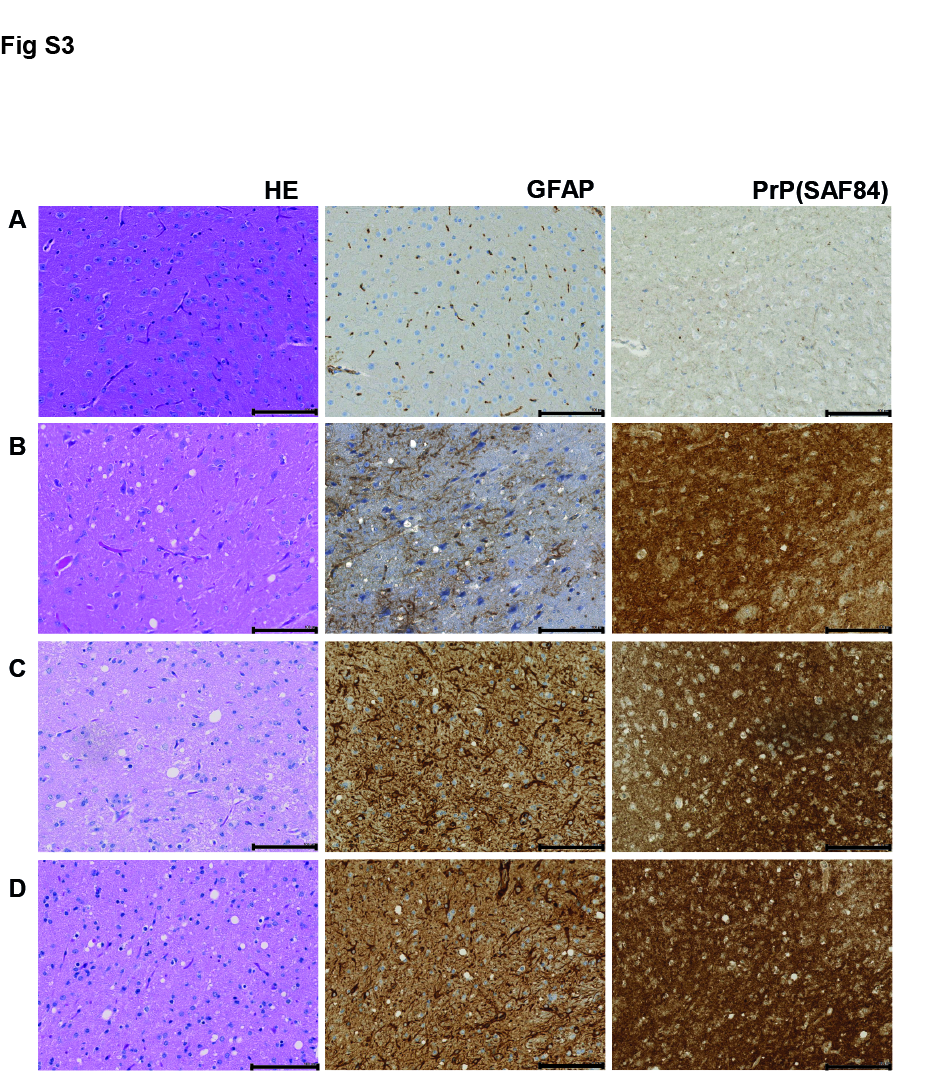

Supplement: S3 Fig — Non-inoculated mice (A) show no evidence of vacuolation, PrPSc deposition or gliosis. Mice inoculated with 263K prion-infected hamster brain homogenate (B), inoculated with PSR1 beads incubated with plasma pools from symptomatic hamster at 117–118 dpi (C) and at 143 and 154 dpi (D) show vacuoles in the HE stained section, PrPc and PrPSc deposition is visualized by the PrP antibody SAF84 and astrocytic gliosis is evidenced by an antibody directed against GFAP. (Scale bars: 100 μm). (TIF) [file pone.0216013.s004.tif]

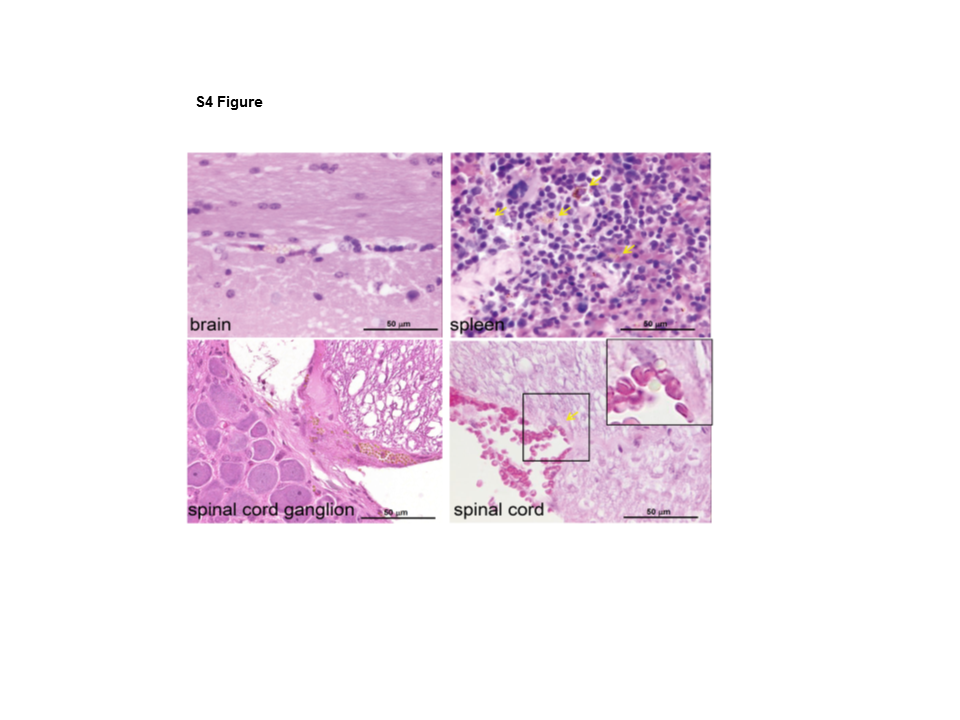

Supplement: S4 Fig — The mouse shown was inoculated with RML6 brain homogenate (10−9 dilution) and euthanized at 253 dpi. HE stained sections of brain, spleen, spinal cord ganglion and spinal cord out nerve are shown. Beads are indicated with yellow arrows. (Scale bars: 50 μm). (TIF) [file pone.0216013.s005.tif]

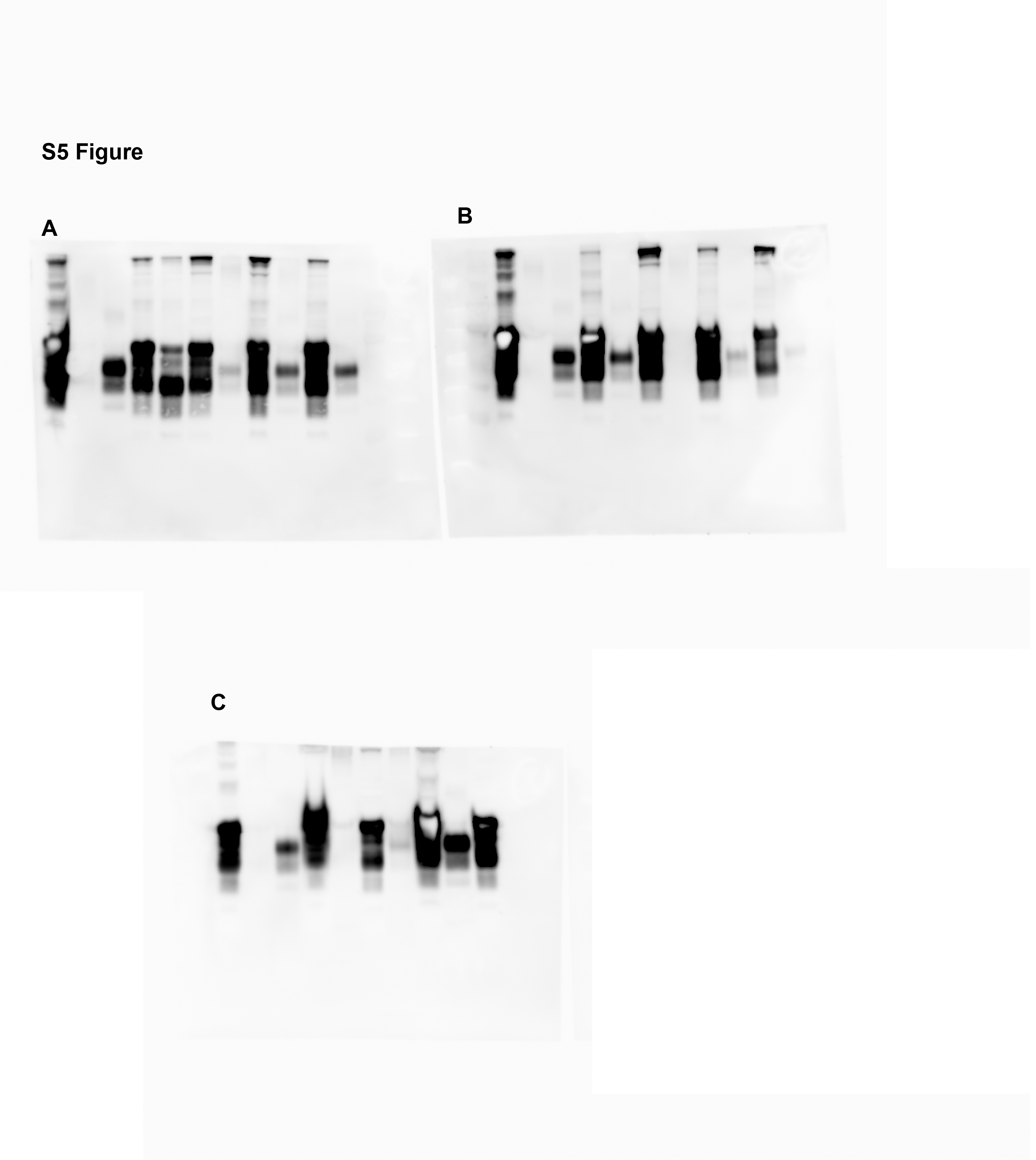

Supplement: S5 Fig — (TIF) [file pone.0216013.s006.tif]
