# Supplementary material for: Enhanced detection of prion infectivity from blood by preanalytical enrichment with peptoid-conjugated beads
Source: PLoS One. 2019 Sep 12;14(9):e0216013. doi: 10.1371/journal.pone.0216013 (PMC6742390; doi:10.1371/journal.pone.0216013)
Supplement: S1 Table — Yes: beads were present in these body fluids. No: no beads were observed. n.a.: non-analyzed. dpi: days post infection. The analysis was only performed for the indicated mice. (PDF) [file pone.0216013.s007.pdf]

S1 Table: Location of PSR1 beads in body fluids from mice inoculated with 3 µl beads coated with plasma from prion-infected hamster per mouse. Yes: beads were present in these body fluids. No: no beads were observed. n.a.: non-analyzed. dpi: days post infection. The analysis was only performed for the indicated mice.

		**body fluids**					
mouse No.	dpi	CSF	blood smear	blood 500-700 µl	urine	feces	gall
1902-1	95	yes	n.a.	n.a.	n.a.	n.a.	n.a.
1902-2	97	yes	n.a.	n.a.	n.a.	n.a.	n.a.
1902-3	100	yes	no	no	n.a.	n.a.	n.a.
1902-4	173	no	no	n.a.	yes	yes	yes
1904B-1	98	yes	n.a.	n.a.	n.a.	n.a.	n.a.
1905-3	100	yes	no	no	n.a.	n.a.	n.a.
1905-4	100	no	no	no	n.a.	n.a.	n.a.
1906-1	96	yes	no	no	n.a.	n.a.	n.a.
1906-2	103	yes	no	no	n.a.	n.a.	n.a.
1906-3	108	yes	no	n.a.	n.a.	n.a.	n.a.
1906-4	116	yes	no	n.a.	n.a.	n.a.	n.a.
1907-1	96	yes	no	no	n.a.	n.a.	n.a.
1907-2	107	yes	no	n.a.	n.a.	n.a.	n.a.
1910-1	70	yes	no	no	n.a.	n.a.	n.a.
1911-1	48	yes	no	n.a.	n.a.	n.a.	n.a.
1911-2	81	yes	no	n.a.	yes	n.a.	yes
1911-3	82	yes	no	n.a.	yes	n.a.	yes
1912-2	37	yes	no	no	n.a.	n.a.	n.a.
1913-1	76	n.a.	n.a.	n.a.	no	yes	n.a.
1913-2	78	yes	no	n.a.	no	yes	n.a.
1913-3	78	yes	no	n.a.	no	yes	n.a.
1913-4	79	yes	no	n.a.	no	yes	n.a.
