# Supplementary material for: Enhanced detection of prion infectivity from blood by preanalytical enrichment with peptoid-conjugated beads
Source: PLoS One. 2019 Sep 12;14(9):e0216013. doi: 10.1371/journal.pone.0216013 (PMC6742390; doi:10.1371/journal.pone.0216013)
Supplement: S2 Table — Yes: beads were found in these homogenates. No: no beads were observed. n.a.: non-analyzed. dpi: days post infection. The analysis was only performed for the indicated mice. (PDF) [file pone.0216013.s008.pdf]

S2 Table: Presence of PSR1 beads in various tissue homogenates from mice inoculated with 3 μl beads coated with plasma from prion-infected hamster per mouse. Yes: beads were found in these homogenates. No: no beads were observed. n.a.: non-analyzed. dpi: days post infection. The analysis was only performed for the indicated mice.

		Homogenates						
mouse No.	dpi	brain	spinal cord	spleen	kidney	lung	liver	heart
1902-1	95	yes	n.a.	yes	n.a.	n.a.	n.a.	n.a.
1902-2	97	yes	n.a.	yes	n.a.	n.a.	n.a.	n.a.
1902-3	100	yes	yes	yes	no	no	no	no
1904B-1	98	yes	n.a.	no	n.a.	n.a.	n.a.	n.a.
1905-1	89	yes	n.a.	no	n.a.	n.a.	n.a.	n.a.
1905-2	91	yes	n.a.	yes	n.a.	n.a.	n.a.	n.a.
1905-3	100	n.a.	no	n.a.	n.a.	n.a.	no	n.a.
1905-4	100	n.a.	no	n.a	n.a.	n.a.	no	n.a.
1906-1	96	yes	n.a.	yes	n.a.	n.a.	n.a.	n.a.
1906-2	103	n.a.	no	n.a.	n.a.	n.a.	no	n.a.
1906-3	108	n.a.	yes	n.a.	no	n.a.	no	no
1906-4	116	n.a.	yes	n.a	no	yes	no	no
1907-1	96	yes	n.a.	yes	n.a.	n.a.	n.a.	n.a.
1910-1	70	yes	n.a.	yes	n.a.	n.a.	n.a.	n.a.
1911-1	48	n.a.	yes	n.a	no	no	yes	n.a.
1912-2	37	n.a.	yes	n.a.	n.a.	n.a.	no	n.a.
