# Supplementary material for: Enhanced detection of prion infectivity from blood by preanalytical enrichment with peptoid-conjugated beads
Source: PLoS One. 2019 Sep 12;14(9):e0216013. doi: 10.1371/journal.pone.0216013 (PMC6742390; doi:10.1371/journal.pone.0216013)
Supplement: S4 Table — Animals were monitored 3 times per week after RML6 or 263K inoculation for clinical signs including gait, grooming, activity, rough hair coat, limb paresis and ataxia. Once the animals showed the first sign of scrapie (grade 1), they were monitored every day and wet food was supplied in the cage. When the animals reached score grade 2 that hindered them reaching the water bottle, they were either euthanized with 3% isofluorane/L O2 (hamster) or methoxyflurane (mice), followed by decapitation. (PDF) [file pone.0216013.s010.pdf]

S4 Table: Clinical assessment and scoring of Golden Syrian hamsters inoculated with the 263K hamster prion strain, *tga*20 mice inoculated with RML6, and *Tg*(SHaPrP) mice either inoculated with 263K or with plasma coated PSR1 beads as described in [1].

Animals were monitored 3 times per week after RML6 or 263K inoculation for clinical signs including gait, grooming, activity, rough hair coat, limb paresis and ataxia. Once the animals showed the first sign of scrapie (grade 1), they were monitored every day and wet food was supplied in the cage. When the animals reached score grade 2 that hindered them reaching the water bottle, they were either euthanized with 3% isofluorane/L O2 (hamster) or methoxyflurane (mice), followed by decapitation.

**Score**	**Clinical signs**	**Assessment**	**Action**
0	No detectable signs of abnormal movement		
1	Waddling gait, mild signs of reduced grooming, limb weakness, front leg paresis	Slight rolling while shaking the cage	Wet food was provided in the cage;<br>Animals were observed every day
2	Ataxia, reduced grooming and activity, paralysis, rough hair coat, rolling	Rolling while shaking the cage	Animals were immediately euthanized once the clinical signs hamper them from reaching the water bottle
3	Dead		

References

1. Zhu C, Schwarz P, Abakumova I, Aguzzi A. Unaltered Prion Pathogenesis in a Mouse Model of High-Fat Diet-Induced Insulin Resistance. PLOS ONE. 2015;10(12):e0144983.
