# Supplementary material for: Enhanced detection of prion infectivity from blood by preanalytical enrichment with peptoid-conjugated beads
Source: PLoS One. 2019 Sep 12;14(9):e0216013. doi: 10.1371/journal.pone.0216013 (PMC6742390; doi:10.1371/journal.pone.0216013)
Supplement: S5 Table — (PDF) [file pone.0216013.s011.pdf]

S5 Table. Mortality of hamster and mice that occurred outside of planned euthanasia or humane endpoints.

**Experiment**	**Animals**
Pilot experiment bead toxicity for bead toxicity	4 *Tga*20 mice died directly after inoculation, because of anesthetics' side effects.<br>1 *Tga*20 mice died at 8 dpi of an intercurrent death.
Inoculation of hamster with 263K prions	No unexpected deaths of hamster were observed.
Bead based PrP^{Sc} capture assays	7 *Tg*SHaPrP mice died directly after inoculation, because of anesthetics' side effects.<br>2 *Tga*20 mice died at 141 and 247 dpi (from total 72 mice) of an intercurrent death.<br>6 TgSHaPrP mice (from total 99 mice) died outside the humane endpoint on scrapie (Score 2, Table S4), because of unexpected fast progression of disease in the last hours.
