# Supplementary material for: Enhanced detection of prion infectivity from blood by preanalytical enrichment with peptoid-conjugated beads
Source: PLoS One. 2019 Sep 12;14(9):e0216013. doi: 10.1371/journal.pone.0216013 (PMC6742390; doi:10.1371/journal.pone.0216013)
Supplement: S3 Table — Paraffin sections of various organs were analysed for the presence of the beads. Yes: beads were found in these organs. No: no beads were observed. n.a.: non-analyzed.?: not clearly identified. BM: bone marrow. dpi: days post infection. The analysis was only performed for the indicated mice. (PDF) [file pone.0216013.s009.pdf]

S3 Table: Presence of PSR1 beads in various organs from mice inoculated with 3 µl beads coated with plasma from prion-infected hamster per mouse. Paraffin sections of various organs were analysed for the presence of the beads. Yes: beads were found in these organs. No: no beads were observed. n.a.: non-analyzed. ?: not clearly identified. BM: bone marrow. dpi: days post infection. The analysis was only performed for the indicated mice.

		paraffin histology										
**mouse No.**	**dpi**	**brain**	**spinal cord**	**spleen**	**kidney**	**lung**	**liver**	**heart**	**pancreas**	**muscle**	**BM**	**gut**
1902-1	95	yes	yes	yes	yes	yes	yes	n.a.	n.a.	n.a.	n.a.	n.a.
1902-2	97	yes	yes	yes	yes	yes	yes	n.a.	n.a.	n.a.	n.a.	n.a.
1902-3	100	yes	yes	yes	yes	yes	yes	yes	no	n.a.	yes	n.a.
1902-4	173	no	no	no	no	no	no	no	no	no	n.a.	n.a.
1904B-1	98	yes	yes	yes	yes	n.a.	n.a.	n.a.	no	n.a.	n.a.	n.a.
1905-1	89	yes	yes	yes	no	yes	no	no	no	n.a.	n.a.	n.a.
1905-2	91	yes	yes	yes	yes	yes	no	no	yes	n.a.	n.a.	n.a.
1905-3	100	yes	yes	yes	yes	no	no	yes	yes	yes	n.a.	n.a.
1905-4	100	yes	yes	no	yes	yes	yes	no	yes	yes	n.a.	n.a.
1906-1	96	yes	yes	yes	yes	no	yes	yes	no	n.a.	n.a.	n.a.
1906-2	103	yes	yes	yes	no	no	yes	yes	no	yes	yes	n.a.
1906-3	108	yes	yes	yes	yes	yes	no	yes	yes	n.a.	n.a.	n.a.
1906-4	116	yes	no	no	no	no	no	no	no	n.a.	n.a.	n.a.
1907-1	96	yes	yes	yes	yes	yes	no	yes	yes	yes	n.a.	n.a.
1907-2	107	yes	no	no	yes	no	yes	no	no	n.a.	n.a.	n.a.
1908-4	253	yes	yes	n.a.	no	no	no	?	no	no	n.a.	n.a.
1909A-1	253	yes	yes	n.a.	no	no	n.a.	?	n.a.	no	n.a.	n.a.
1909A-2	253	yes	yes	n.a.	no	no	no	?	n.a.	no	n.a.	n.a.
1909B-1	253	yes	yes	n.a.	no	no	no	?	n.a.	no	n.a.	n.a.
1909B-2	253	yes	yes	n.a.	no	?	no	?	n.a.	no	n.a.	n.a.
1910-1	70	yes	yes	yes	yes	yes	yes	yes	yes	yes	n.a.	n.a.
1911-1	48	yes	no	no	yes	yes	no	yes	no	n.a.	n.a.	n.a.
1912-2	37	yes	yes	yes	yes	yes	yes	no	yes	yes	n.a.	n.a
1913-1	76	yes	no	yes	yes	yes	yes	n.a.	yes	yes	n.a.	no
1913-2	78	yes	n.a.	n.a.	n.a.	n.a.	n.a.	n.a.	n.a.	n.a.	n.a.	no
